# Dim and Small Target Detection with a Combined New Norm and Self-Attention Mechanism of Low-Rank Sparse Inversion

**DOI:** 10.3390/s23167240

**Published:** 2023-08-18

**Authors:** Lei Min, Anqing Wu, Xiangsuo Fan, Feng Li, Juliu Li

**Affiliations:** 1 Institute of Optics and Electronics, Chinese Academy of Sciences, Chengdu 610209, China; minlei@ioe.ac.cn (L.M.); lifeng@ioe.ac.cn (F.L.); 2 Key Laboratory on Adaptive Optics, Chinese Academy of Sciences, Chengdu 610209, China; 3 School of Automation, Guangxi University of Science and Technology, Liuzhou 545006, China; 221055210@stdmail.gxust.edu.cn (A.W.); 221055176@stdmail.gxust.edu.cn (J.L.); 4 Guangxi Collaborative Innovation Centre for Earthmoving Machinery, Guangxi University of Science and Technology, Liuzhou 545006, China

**Keywords:** infrared target detection, linear transformation, new norm, self-attention mechanism, alternating direction multiplier method

## Abstract

Methods for detecting small infrared targets in complex scenes are widely utilized across various domains. Traditional methods have drawbacks such as a poor clutter suppression ability and a high number of edge residuals in the detection results in complex scenes. To address these issues, we propose a method based on a joint new norm and self-attention mechanism of low-rank sparse inversion. Firstly, we propose a new tensor nuclear norm based on linear transformation, which globally constrains the low-rank characteristics of the image background and makes full use of the structural information among tensor slices to better approximate the rank of the non-convex tensor, thus achieving effective background suppression. Secondly, we construct a self-attention mechanism in order to constrain the sparse characteristics of the target, which further eliminates any edge residuals in the detection results by transforming the local feature information into a weight matrix to further constrain the target component. Finally, we use the alternating direction multiplier method to decompose the newly reconstructed objective function and introduce a reweighted strategy to accelerate the convergence speed of the model. The average values of the three evaluation metrics, SSIM, BSF, and SNR, for the algorithm proposed in this paper are 0.9997, 467.23, and 11.72, respectively. Meanwhile, the proposed detection method obtains a higher detection rate compared with other algorithms under the same false alarm rate.

## 1. Introduction

The weak target detection algorithm in infrared is an important technology for infrared warning and guidance. In real-world scenarios, the imaging distance of the target is far, the imaging area is small, and the radiation energy is low, which makes the target easily affected by noise and clutter interference. Therefore, how to separate weak targets from complex backgrounds is a challenging research topic, and many scholars have conducted in-depth research on this issue [1,2].

Traditional spatial–temporal filtering methods are one of the mainstream weak target detection algorithms in infrared, which separate the target and the background by local feature information of the image in the spatial–temporal domain. There are two methods of temporal filtering: single-frame detection algorithms and multi-frame detection algorithms. Multi-frame detection algorithms are mainly three-dimensional matching filtering algorithm, projection transformation algorithm, dynamic programming algorithm, pipeline filtering algorithm, etc. Common single-frame detection algorithms include top-hat filtering algorithm, gradient reciprocal filtering algorithm, etc. The principle of three-dimensional matching filtering is to set a three-dimensional filter on all possible motion trajectories of the target, and then analyze and judge the collection results of the filter. The three-dimensional filter with the highest signal-to-noise ratio is determined as the weak target and motion trajectory. However, for motion-mismatched targets, multiple velocity filters need to be set to capture the target trajectory, which leads to an increase in the computational complexity of the detection algorithm [3]. The projection transformation projects multidimensional data into a low-dimensional space, and obtain the motion trajectory of the target from the low-dimensional data. Since this algorithm appears to reduce the dimensionality of the data during the calculation, the data of the samples to be processed and the suspicious target signals are greatly reduced, which simplifies the computation. Compared with the multidimensional matching filtering, reduced computation is accompanied by poorer target detection result [4,5]. In the study of small target detection, Wang et al. [6] first used feature triangles to perform registration on infrared images and reduced the computation by using the star point coordinate matrix method. Then, the maximum value projection transformation method was used to process the sequence images and, therefore, obtain the result. The idea based on dynamic programming is to equivalently treat the accumulated energy of the detected small target on a certain trajectory as the decision function in the theory of dynamic programming. The motion range of the target at different stages is regarded as the decision space. Then, by recursive methods, the motion trajectory that can make the decision function achieve global optimization is found [7]. Guo et al. [8] proposed a dynamic programming method based on parallel computing, which performs a parallel search on the suspicious candidate target area to obtain the candidate target points. This method effectively improves the detection efficiency, but its detection performance decreases as the target signal-to-noise ratio gradually decreases. The principle of the pipeline filtering algorithm is that when the target signal is sampled at an appropriate frequency, the motion characteristics of the target signal in space are continuous in time, while random noise does not have continuity. Dong et al. [9] proposed a method that combines a modified visual attention model (VAM) with anti-jitter pipeline filtering algorithm. In this method, the best mode is adaptively selected to calculate the saliency map. Then, a local saliency singularity evaluation strategy is designed to automatically extract suspicious targets and suppress background clutter. Finally, the real target is distinguished by anti-vibration pipe filtering. This method can improve the efficiency of target detection in infrared imaging under different weather conditions. Although many improved pipeline methods have been proposed, it is difficult to achieve good detection performance when the motion of small targets has complex and varied characteristics. The top-hat filtering algorithm models the background of the image by using a structural element as a template for opening and closing operations on the image, and obtains the final difference map. Bai et al. [10] combined multi-scale operations with structural elements, which predict the image background by different scale structural elements. The top-hat filtering algorithm causes a loss of target energy intensity during opening and closing operations, resulting in poor detection in scenes with low target energy. Li et al. [11] established a correlation coefficient function based on the local feature information of the image, and introduced it into the gradient reciprocal filter algorithm, which can obtain an improved background estimation. Due to the complex and variable gray-scale characteristics of the image background, the filtering template of the gradient reciprocal filter algorithm cannot completely match the background features, resulting in a large number of edge residues in the detection results. Most real-world scenarios contain a lot of noise and clutter, and the noise signal is only slightly different from the weak target in terms of texture and energy. It is difficult to fully distinguish noise from the target based on only local feature information in the image. Therefore, traditional spatio-temporal filtering algorithms are easily interfered by noise and clutter, leading to low detection rates.

In the study of infrared faint target detection with deep learning, in order to effectively improve the generalization ability of the deep neural network detection model, Dai et al. proposed a special ACM detection model based on the construction of a high-quality annotation data set. There is an asymmetric upper and lower information modulation model for small infrared target detection [12]. This model not only realizes forward and backward information transfer to fusion, but also supplements a top-down information modulation module based on point-oriented channel attention, which enhances the fusion of target signals. The DNAnet network proposed by Li et al. completes the feature fusion of the target through the repeated use of image features [13], making full use of the weak information of the target. In addition, the model also includes the constructed dense interaction module and channel-attention module, which realizes the fusion of target features and the enhancement of adaptive features, and has achieved remarkable results in the detection of small infrared targets. In order to solve the problems of detection loss (MD) and false alarm (FA) more effectively, Wang et al. proposed an adversarial network consisting of two generators and one discriminator to extract target feature information [14]. This model distributes detection tasks to different training models, which improves the overall detection efficiency of the model. To sum up, the weak and small target detection model of deep learning has achieved remarkable results, but considering the limitations of the generalization ability of the model brought about by the multiple changes in the air and space detection environment, it is necessary to start from the data set construction and model construction. From the perspective of research, increase the practical application of deep learning detection models in the detection of small and weak infrared targets.

In recent years, scholars have paid more attention to the information about the entire image. They have transformed the problem of detecting targets into the matrix reconstruction problem through machine learning theories, and solved this problem using the overlapping direction multiplier method to obtain the detect result. Gao et al. [15] proposed an infrared patch-image (IPI) model that utilizes the correlation characteristics of non-local image background. Wang et al. [16] suppressed strong background edge contours by combining anisotropy and L1 norm on the basis of the IPI model. Xue et al. [17] suppressed strong background edge contours by combining anisotropy and the L1 norm. Xue et al. used the weighted L1 norm to constrain the target and added constraints on the noise using L1,1 and L2,1 norms to eliminate the background residue. However, in a complex background, the simple nuclear norm cannot fully constrain the background. These methods are unable to fully recover the background image, causing unsatisfactory detection results. Compared with two-dimensional data, high-dimensional data have more structural information, making it easier for us to explore hidden relationships between data. Therefore, Dai et al. [18] proposed an infrared patch tensor (IPT) model to extend the two-dimensional image matrix to three-dimensional, fully exploring the spatial correlation of non-local areas of the image, and using the prior information to constrain sparse targets through re-weighting. Zhang et al. [19] further suppressed the background using partial sum of the tensor nuclear norm (PSTNN), and then used the corner descriptor function and the background descriptor function to obtain prior information of the image to jointly constrain the target using the L1 norm. Wang et al. [20] constructed a new spatio-temporal tensor model, non-overlapping patch spatial–temporal tensor (NPSTT) model, based on the IPT model to avoid redundant information. The method also obtains better target constraint results with the help of tensor upper bound nuclear norm. Kong et al. [21] approximated the non-convex tensor fiber rank using Log tensor fibered nuclear norm (LogTFNN) on the basis of the IPT model, and then used hypertotal variation (HTV) to suppress noise. Cao et al. [22] defined a mode-k1k2 extension tensor tubal rank (METTR) and fully preserve the spatial correlation of non-local areas in images by METTR-norm.

During the actual process of target detection, the background is complex, often accompanied by strong edge contours and strong noise. Merely using the information about the entire image cannot completely suppress the strong edges, leading to residual background in the detection results. The IPT model constructs background weight function based on the characteristics of the structural tensor, but ignores the information of the target, leading to excessive contraction of the target. The PSTTN model proposes a structural tensor descriptor function for both the background and the corner points based on the RIPT model, while retaining the prior information of both the background and the target. However, because the prior information related to the background is only based on the maximum value of the eigenvalues, it cannot completely suppress the contours of strong edge contours, resulting in some edge contours being retained in the final local feature prior information.

In summary, in order to solve the limitations of existing detection models, we propose a low-rank sparse inversion method for detecting small targets by combining a new norm with self-attention mechanisms.

1. To better constrain the low-rank tensor, we approximate the tensor rank using a new tensor nuclear norm that is non-convex. The new tensor nuclear norm preserves the structural information between the various slices of the tensor and can accurately recover low-rank backgrounds under certain interference. Moreover, the high-order tensor is reduced during the operation, reducing the computational complexity of the algorithm.
2. We construct a self-attention mechanism model to constrain the sparse characteristics of the target. This model obtains local feature information of the image to suppress strong edge contours and strong noise and transforms the local feature information into a weight matrix to introduce the constraint of the sparse component of the infrared small target detection model.
3. We design an overlapping direction multiplier method to solve the infrared small target detection model and obtain the target image. In the solving process, we use a re-weighting strategy to accelerate the convergence speed and accuracy of the model.

## 2. Introduction to the Principle of Tensor Singular Value Decomposition (T-SVD) [23]

We define a invertible linear transformation $L:R^{n_{1}\times n_{2}\times n_{3}}\to R^{n_{1}\times n_{2}\times n_{3}}$, for any invertible transformation *L*, we have $L\left(0\right)=L^{-1}\left(0\right)=0$, and obtaining $\bar{A}$ by linearly transforming A along the third dimension, as shown in:

(1) $$\bar{A}=L\left(A\right)=A\times_{3}L.$$

The symbol $\times_{3}$ in the formula represents the mode-3 product. The linear transformation $L\in R^{n_{3}\times n_{3}}$ is any invertible matrix. Under any invertible linear transformation *L*, the singular value decomposition of $\bar{A}\in R^{n_{1}\times n_{2}\times n_{3}}$ is given by:

(2) $$\left[{\bar{U}}^{\left(i\right)},{\bar{S}}^{\left(i\right)},{\bar{V}}^{\left(i\right)}\right]=SVD\left({\bar{A}}^{\left(i\right)}\right).$$

In the above equation ${\bar{U}}^{\left(i\right)},{\bar{S}}^{\left(i\right)},{\bar{V}}^{\left(i\right)},{\bar{B}}^{\left(i\right)}\in R^{n_{1}\times n_{2}}$ denote the i-th $\left(i=1\cdots n_{3}\right)$ frontal slice of $U,S,V,A\in R^{n_{1}\times n_{2}\times n_{3}}$, respectively. Where $U\in R^{n_{2}\times n_{2}\times n_{3}}$ and $V\in R^{n_{2}\times n_{2}\times n_{3}}$ are the orthogonal tensor, $S\in R^{n_{2}\times n_{2}\times n_{3}}$ is the diagonal tensor. The inverse mapping of ${\bar{U}}^{\left(i\right)},{\bar{S}}^{\left(i\right)},{\bar{V}}^{\left(i\right)}$ are given by

(3) $$\left\{\begin{array}{c} U=L^{-1}\left(\bar{U}\right)=\bar{U}\times_{3}L^{-1}\\ S=L^{-1}\left(\bar{S}\right)=\bar{S}\times_{3}L^{-1}\\ V=L^{-1}\left(\bar{V}\right)=\bar{V}\times_{3}L^{-1}. \end{array}\right.$$

**Figure 1:**
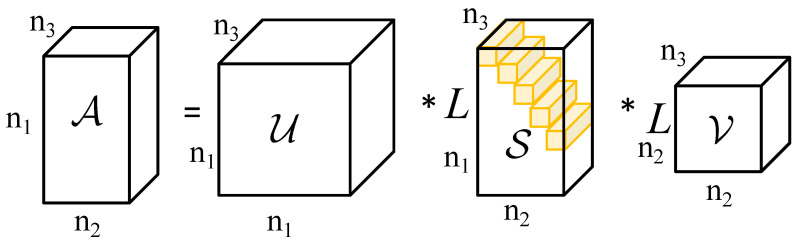
An illustration of the t-SVD under linear transform *L* of $n_{1}\times n_{2}\times n_{3}$.

Figure 1 and Algorithm 1 demonstrate the process of tensor singular value decomposition. As S and $\bar{S}$ are F-diagonal tensors, we can define the tensor tube rank using their non-zero tube counts [24].

**Algorithm 1** T-SVD under linear transform *L***Input:**$A\in R^{n_{1}\times n_{2}\times n_{3}}$ and invertible linear transform *L*;**Step 1.** Compute $\bar{A}=L\left(A\right)$;**Step 2.** Frontal slicing of image $\bar{A}$ and singular value decomposition of each frontal slice combined with Equation (2) to obtain $\bar{U}$, $\bar{S}$ and $\bar{V}$**Step 3.** The inverse linear transformation of the three tensors is calculated by Equation (3) to obtain U, S and V
**Output:**

U,S,V



## 3. NNSAM Model

### 3.1. Constructing New Tensor Nuclear Norm Constrained Background Low Rank Properties

In the absence of noise, the infrared weak small target detection model can be transformed into a Tensor Robust Principal Component Analysis (TRPCA) model, which expression is:

(4) $$\begin{array}{c} \arg\min_{A,E}{∥A∥}_{*}+\lambda {∥E∥}_{1}\\ s.t.\;D=A+E, \end{array}$$

where D,A,E represent the original image tensor of the sequence, the background image tensor of the sequence, and the target image tensor of the sequence, respectively. λ is a positive parameter. Considering that the TRPCA model mentioned above is disrupted by the strong edge contours and noise in the environment with many strong noise points, causing the bad detection result, we have made improvements to the above model. Firstly, we introduce the tensor nuclear norm based on linear transformation to replace the original tensor nuclear norm, which constrains the image background globally. We use Algorithm 1 based on linear transformations of tensor singular value decomposition and tensor t-product to define tensor spectral norm, and then derive the new tensor nuclear norm by the property of tensor spectral norm as the dual norm of tensor nuclear norm. The definition of the new tensor nuclear norm is as follows [25,26]:

(5) $${∥A∥}_{*}=\frac{1}{l}{∥\bar{A}∥}_{*},$$

where l>0 is a constant. $A\in R^{n_{1}\times n_{2}\times n_{3}}$, $\bar{A}\in R^{n_{1}n_{3}\times n_{2}n_{3}}$ are the block diagonal matrix, and its i-th block on the diagonal is the i-th slice ${\bar{A}}^{\left(i\right)}$ of $\bar{A}$. The new tensor nuclear norm is able to approximate non-convex tensor rank well, while preserving the structural information between each tensor slice, and achieving dimensionality reduction for high-order tensors with low time complexity and high computational efficiency. Therefore, this paper adopts the new tensor nuclear norm to build the detection model. The model expression of the new tensor nuclear norm is as follows,

(6) $$\begin{array}{c} \underset{A,E}{\arg\min}\frac{1}{l}{∥\bar{A}∥}_{*}+\lambda {∥E∥}_{1}\\ s.t.\;D=A+E, \end{array}$$

### 3.2. Constructing Self-Attention Mechanisms to Constrain the Sparsity of the Target Feature

The detection model constructed based on non-local feature information of images can well recognize complex edge contours in the background of images. However, it cannot completely suppress some strong edge contours and strong noise points, lead to residual edge contours and noise in the detection results. Therefore, many scholars use traditional filtering or human visual saliency methods to obtain local feature information of images, and then convert the local feature information into a weight matrix and introduce it into the infrared weak small target detection model constructed by non-local feature information. Scholars have found that in infrared images containing weak targets, the high-order singular values obtained through SVD decomposition usually reflect the noise in the image, the middle-order singular values reflect the targets in the infrared image, and the low-order singular values reflect the background in the infrared image [27]. Based on these characteristics, the singular value decomposition of the image matrix in this article can achieve background suppression. Assuming that the rank of matrix $X\in R^{m\times n}$ is r≤min(m,n), the singular value decomposition of matrix t can be expressed as follows

(7) $$X=U\sum V^{T}.$$

In the above equation, $U\in R^{m\times m}$ and $V\in R^{n\times n}$ are orthogonal matrices, which are, respectively, called left and right singular value vector matrices, and $\sum \in R^{m\times n}$ is the singular value matrix. Since the high-order and low-order singular values reflect the noise and background of the infrared image, respectively, the target image can be obtained by directly truncating the high-order and low-order singular values of the singular value matrix through the double threshold method [27]. Assuming that the truncated singular value matrix is $\sum^{\prime }$, the SVD filtering result can be calculated by the following equation

(8) $$X=U\sum^{\prime }V^{T}.$$

**Figure 2:**
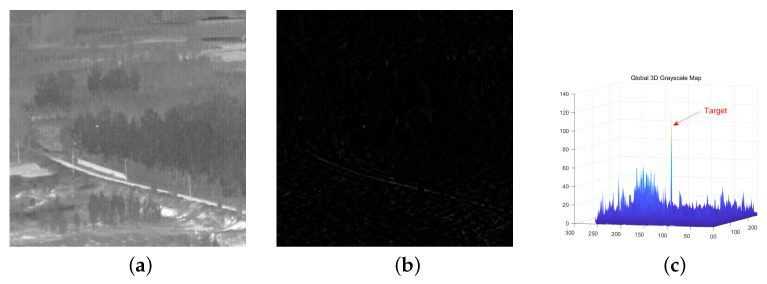
(**a**–**c**) represent the original image, the result of SVD filtering, and the global three-dimensional diagram of the SVD filtering result, respectively.

The result of the singular value filtering obtained through the above thoughts is shown in Figure 2b, and its global three-dimensional diagram is shown in Figure 2c.

From Figure 2, we can see that using the double threshold rule alone cannot accurately determine the high-order and low-order singular values of the infrared image matrix, which leads to the retention of a large number of edge contours and noise residues in the detection results. Therefore, we have developed a target sparse constraint algorithm based on self-attention mechanism to further suppress the edge contours and noise residues in the SVD filtering results.

In cognitive science, due to the bottleneck of information processing, humans often focus on important information while ignoring unimportant information, a mechanism called the self-attention mechanism. The Self-attention mechanism usually focuses on important information with high weights and ignores irrelevant information with low weights.

Moreover, the weights can be continuously adjusted to obtain important information under different circumstances [28]. The core formula of the self-attention mechanism is:

(9) $$Attention\left(Q,K,V\right)=Softmax\left(\frac{QK^{T}}{\sqrt{d_{k}}}\right)V,$$

where Q,K,V represent the Query, Key, and Value matrices, respectively. $d_{k}$ denotes the dimension size of matrix Key, and Softmax is the normalization function. From analyzing the information of the filtered image, it can be seen that there are only a few edge contours and a small amount of noise residues present in the image after filtering. We replace Q,K,V with non-overlapping infrared block image matrices $X_{t}$, the transpose of non-overlapping infrared block image matrices $X_{t}^{T}$, and non-overlapping infrared block image matrices $X_{t}$, respectively. Since the highest correlation between $X_{t}$ and $X_{t}^{T}$ is in the target area, we can achieve target extraction and suppression of edge contours through the idea of self-attention mechanism. The formula established in this paper based on the self-attention mechanism for non-overlapping infrared image blocks is as follows:

(10) $$Attention\left(X_{t}\right)=Softmax\left(X_{t}*X_{t}^{T}\right)⊙X_{t}.$$

where $X_{t}$ denotes the non-overlapping infrared block image matrix, * and ⊙ denote the multiplication of the matrix and the hadamard product of the matrix, respectively, and Softmax() is the normalized function. We found that the background residues in the filtered results always appear as stripes, while the targets appear as dots. Using block image matrices $X_{t}*X_{t}^{T}$, we can enhance the energy of the targets and suppress edge contours. We then normalize the results of the block matrix $X_{t}*X_{t}^{T}$ to use them as weight matrices to constrain the original block image matrix, thereby suppressing the background edge residues. The process of constructing a self-attention mechanism to constrain the sparse characteristics of the target is shown in Algorithm 2.

**Algorithm 2** Target sparse constraint algorithm based on self-attention mechanism**Input:** The result $X\in R^{m\times n}$ of SVD filtering;**Step 1.** The image matrix *X* is divided into block image matrices $X_{p}$ using a sliding window;**Step 2.** The operation result $X_{{}_{p}}^{{}^{\prime }}$ of the self-attention mechanism is obtained by calculating each block image matrix using Equation (10);**Step 3.** The final result matrix $X^{{}^{\prime }}$ is obtained by concatenating the operation result block matrices $X_{{}_{p}}^{{}^{\prime }}$ of the self-attention mechanism;**Output:** The result $X^{{}^{\prime }}$ of the target sparse constraint algorithm after applying the self-attention mechanism.

We construct the tensor $W_{x}\in R^{m\times n\times t}$ by stacking together the results $X^{\prime }$ of t-frame sequence images after the self-attentive mechanism, and then take the inverse of all elements of the tensor $W_{x}$ to obtain $W_{l}$. To accelerate the convergence speed and improve the accuracy of the model, paper [29] proposed a re-weighting strategy which adds a coefficient weight in the model. The expression for this coefficient weight is:

(11) $$W_{sw}^{k+1}=\frac{c}{E^{k}+v},$$

where c represents a positive constant and vs. represents a very small constant to prevent the denominator from being 0. By combining the re-weighting strategy with local feature tensors, we obtain the final local feature tensor as follows:

(12) $$W=W_{l}⊙W_{sw},$$

the objective function of the final model is:

(13) $$\begin{array}{c} \underset{A,E}{\arg\min}\frac{1}{\ell }{∥\bar{A}∥}_{*}+\lambda {∥W⊙E∥}_{1}\\ s.t.\;D=A+E. \end{array}$$

### 3.3. Solving of the Model

For the model represented by Equation (13), we usually use the overlapping direction multiplier method to solve it [30]. The augmented Lagrangian function for Equation (13) is:

(14) $$L\left(A,E,Y,\mu \right)={∥A∥}_{*}+\lambda {∥W⊙E∥}_{1}+\langle Y,A+E-D\rangle +\frac{\mu }{2}{∥A+E-D∥}_{F}^{2},$$

where Y denotes the Lagrangian multiplier, $\langle •\rangle$ denotes the inner product of the tensor, and μ>0 denotes the penalty factor. We divide the augmented Lagrangian function into several sub-problems for solving. With regard to the sub-problem of A, in *k* + 1 iterations,

(15) $$A^{k+1}=\underset{A}{\arg\min}{∥A∥}_{*}+\frac{\mu^{k}}{2}{∥A+E^{k}-D+\frac{Y^{k}}{\mu^{k}}∥}_{F}^{2}.$$

For the above sub-problem, we use the Tensor Singular Value Thresholding (T-SVT) operator [25] for solving. Let $\left(D-E^{k}-\frac{Y^{k}}{\mu^{k}}\right)=U{*}_{L}S{*}_{L}V^{T}$ be tensor SVD of $D-E^{k}-\frac{Y^{k}}{\mu^{k}}$,

(16) $$A^{k+1}=U{*}_{L}S_{1}{*}_{L}V^{T},$$

where $S_{1}=L^{-1}\left({\left(L\left(S\right)-1\right)}_{+}\right)$, here $t_{+}=\max\left(t,0\right)$. With regard to the sub-problem of E, in *k* + 1 iterations,

(17) $$E^{k+1}=\underset{E}{\arg\min}\lambda {∥E⊙W∥}_{1}+\frac{\mu_{k}}{2}{∥A^{k+1}+E-D+\frac{Y^{k}}{\mu_{k}}∥}_{F}^{2},$$

**Figure 3:**
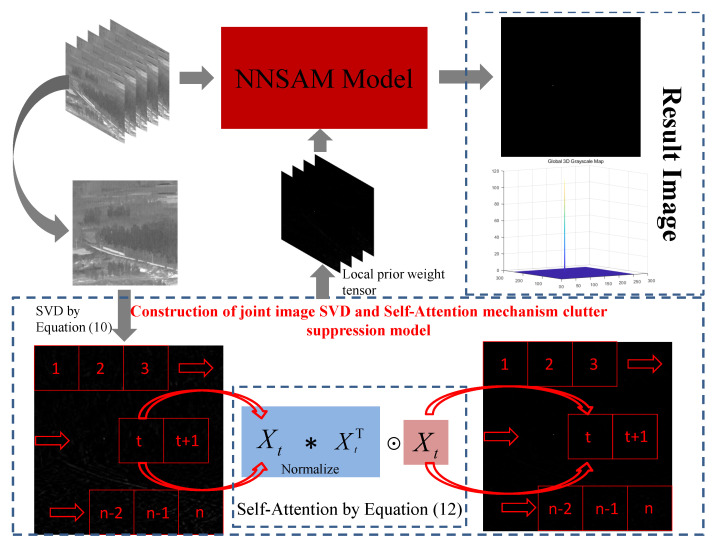
Flow chart of the proposed model.

For the above sub-problem, we use the threshold shrinkage operator for solving:

(18) $$E^{k+1}=S_{\frac{\lambda W^{k}}{\mu_{k}}}\left(D-A^{k+1}-\frac{Y^{k}}{\mu_{k}}\right).$$

where $S_{\tau }\left(X\right)=sign\left(X\right)\max\left(\left|X\right|-\tau ,0\right)$, and sign(•) denotes the sign function. With regard to the sub-problem of Y,μ, in *k* + 1 iterations,

(19) $$\left\{\begin{array}{c} Y^{k+1}=Y^{k}+\mu^{k}\left(A^{k+1}+E^{k+1}-D\right)\\ \mu^{k+1}=\min\left(\rho \mu^{k},\mu_{\max}\right), \end{array}\right.$$

where $\rho ,\mu_{\max}$ are given constants. Finally, Algorithm 3 shows the entire process of the ADMM algorithm, and Figure 3 shows the entire process of the model.

**Algorithm 3** Solve proposed model by ADMM**Input:** tensor data D,W, parameter λ;**Initialization:**$A_{0}=Y_{0}=E{}_{0}=0,\max\_iter=500,\rho =1.1,\mu_{0}=1\times 10^{-3},\mu_{\max}=1\times 10^{10}$, $\delta =1\times 10^{-8},k=0$;while not converge do**Step 1.** Update $A^{k+1}$ by Equation (16);**Step 2.** Update $E^{k+1}$ by Equation (17);**Step 3.** Update $Y^{k+1},\mu^{k+1}$ by Equation (19);**Step 4.** Check the convergence conditions:${∥A^{k+1}-A^{k}∥}_{\infty }\leq \delta$, ${∥E^{k+1}-E^{k}∥}_{\infty }\leq \delta$, ${∥A^{k+1}+E^{k}-D∥}_{\infty }\leq \delta$, k≤max_iter**Step 5.** k:k=k+1end while
**Output:**

B,E



## 4. Experiment and Analysis

**Figure 4:**
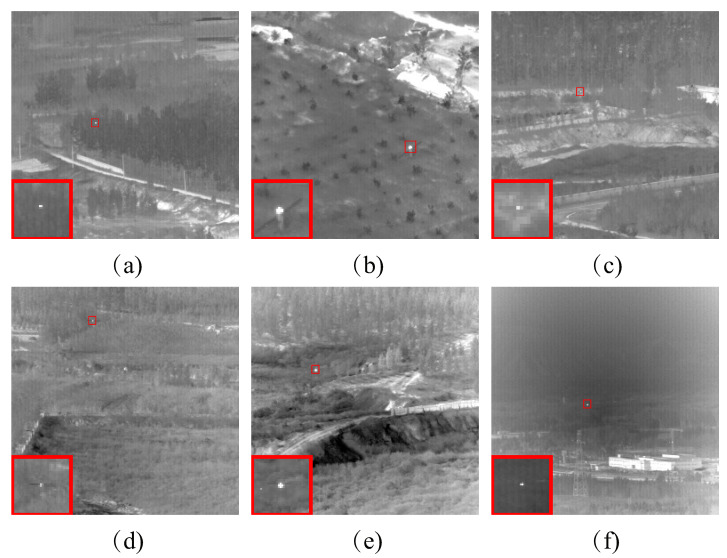
The representative image of the six sequential images. (**a**–**f**) represent the images of six different sequence scenes, respectively.

In this section, we will further showing the advantages of our model from target detection ability, robustness to noise, and adaptability to multiple scenes. We will compare our algorithm with nine advanced algorithms in the weak object detection field on six sequences representing image detection effects [31]. We will also evaluate our algorithm using performance metrics such as SSIM, BSF, SNR, and ROC curves [32]. Figure 4 shows the six sequential images used in the experiments.

### 4.1. Parametric Analysis

In our proposed methods, the size of image patches in the sparse target-constrained algorithm based on the self-attention mechanism, as well as the regularization factor λ and μ in the infrared weak small target detection model, have a significant impact on the algorithm proposed in this paper. Therefore, in order to demonstrate the impact of parameters on the algorithm, we conducted separate analysis on these three parameters. The analysis results are as follows.

#### 4.1.1. Patch Size

**Figure 5:**
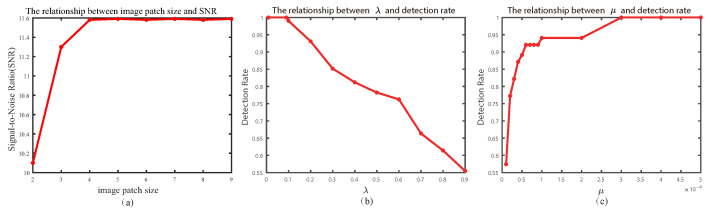
(**a**) represents the relationship between image patch size and SNR, (**b**) represents the relationship between λ and detection rate, and (**c**) represents the relationship between μ and detection rate.

In the background-constrained algorithm based on self-attention mechanism, the size of image patches has a significant impact on the constraint effect of edge contours. Moreover, the size of image patches is related to the size of the target. When the target is larger, the size of image patches should also be larger. Figure 5a shows the relationship curve between the size of image patches and the global signal-to-noise ratio under sequence (a). In this paper, the size of image patches is 5.

#### 4.1.2. Regularization Factor λ

λ is a regularization factor used to balance the sparse target components and low-rank background components in the model. When λ is too small, it can lead to incomplete separation of the low-rank components, resulting in more background edge contours in the detection results. When λ is too large, it can cause the targets to shrink excessively, affecting the detection performance of the model. Therefore, the value of λ has a significant impact on the model, and finding the best balance is a challenge. Figure 5b shows the relationship curve between the value of λ and the detection rate under sequence (a). In this paper, the regularization factor λ is 0.1.

#### 4.1.3. Penalty Factor μ

The penalty factor μ is used to balance the T-SVT operator and the threshold shrinkage operator. The penalty factor can affect the convergence speed and solution accuracy of the model. When the penalty factor is too small, it will lead to excessive shrinkage of the target, resulting in a low detection rate. When the penalty factor is too large, it will cause inaccurate solution results, leading to false positives and false negatives. Figure 5c shows the relationship curve between the value of μ and the detection rate under sequence (a). In this paper, the penalty factor μ is $3\times 10^{-4}$.

### 4.2. Results of Background Suppression Experiments

**Figure 6:**
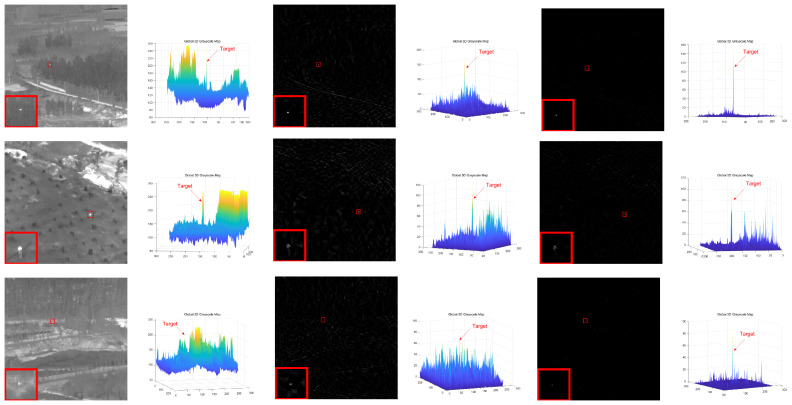

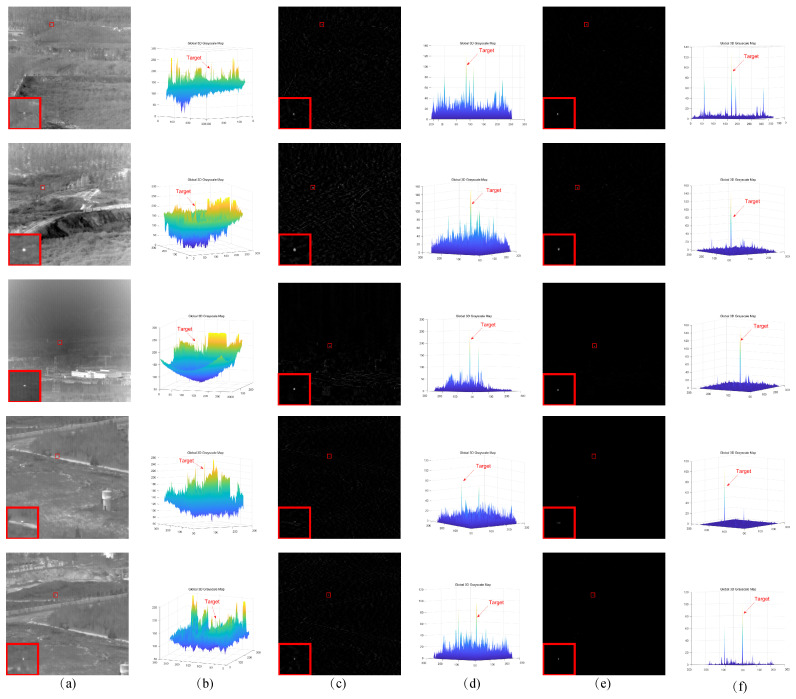
The result of target sparse constraint algorithm based on self-attention mechanism. (**a**,**c**,**e**) represent the original image, SVD filtering result and the result after being constrained by the self-attention mechanism, respectively. (**b**,**d**,**f**) represent the three-dimensional view of the original image, the three-dimensional view of the SVD filtering result, and the three-dimensional view of the result after being constrained by the self-attention mechanism, respectively.

To demonstrate the sparsity constraint effect of the self-attention mechanism algorithm proposed in this paper, we conducted experiments on the eight scenes and the experiments result are shown in Figure 6. The results suggested that the self-attention mechanism-based sparsity constraint algorithm can effectively suppress edge residual and noise in the background by dividing the image matrix into blocks and performing the operation in Equation (10), achieving good background suppression effect. Figure 6a,c,e, respectively, represent the original image, SVD filtering result and the result after being constrained by the self-attention mechanism, while Figure 6b,d,f, respectively, represent the three-dimensional view of the original image, the three-dimensional view of the SVD filtering result, and the three-dimensional view of the result after being constrained by the self-attention mechanism.

### 4.3. Visual Comparison with Baselines

**Figure 7:**
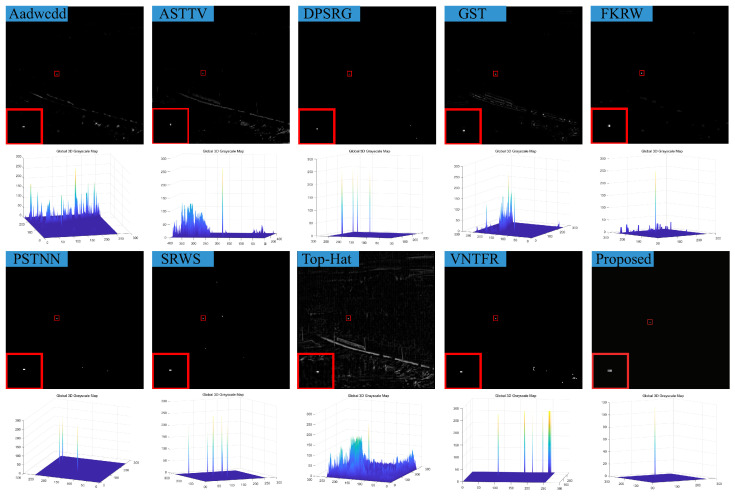
Visual detection effects of compared methods in scene 1.

**Figure 8:**
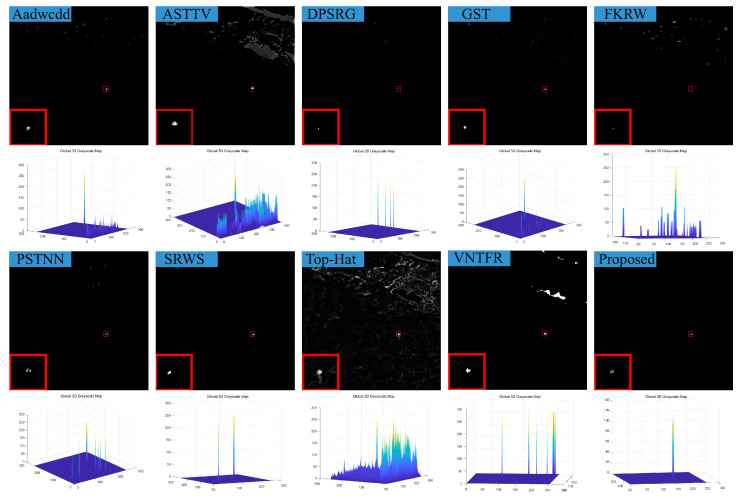
Visual detection effects of compared methods in scene 2.

**Figure 9:**
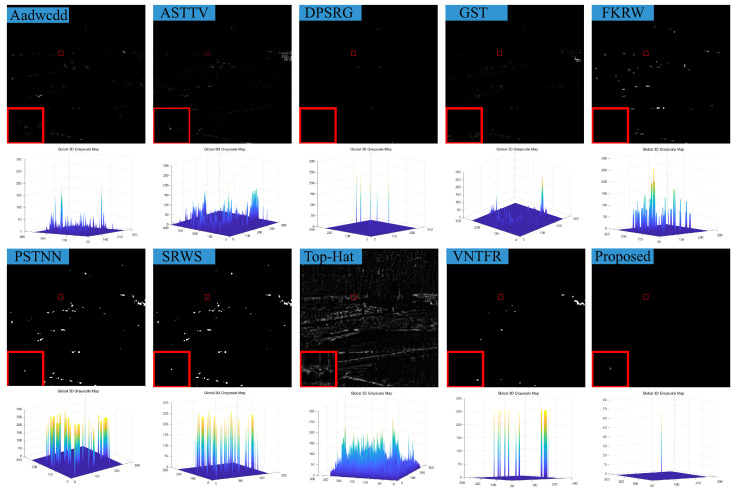
Visual detection effects of compared methods in scene 3.

**Figure 10:**
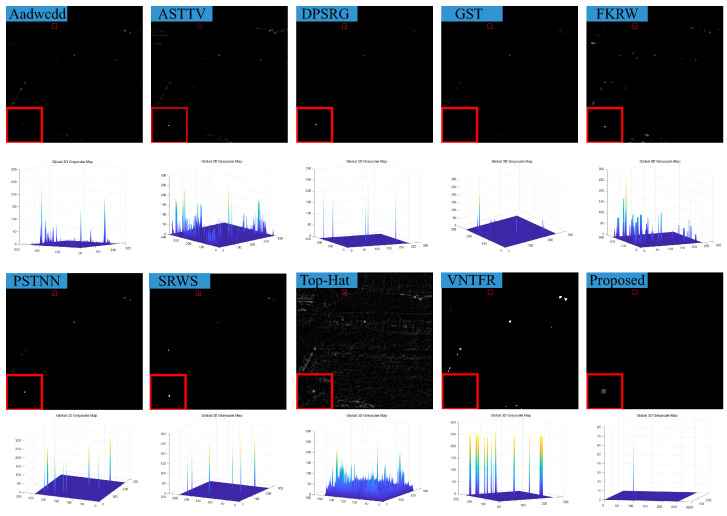
Visual detection effects of compared methods in scene 4.

**Figure 11:**
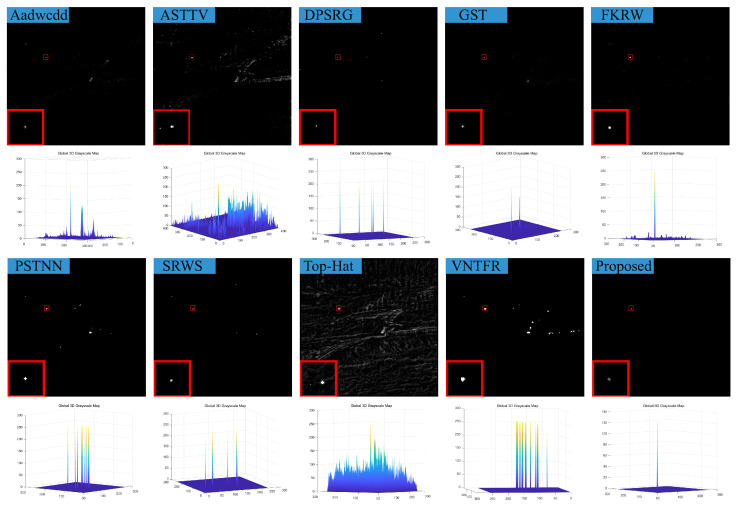
Visual detection effects of compared methods in scene 5.

**Figure 12:**
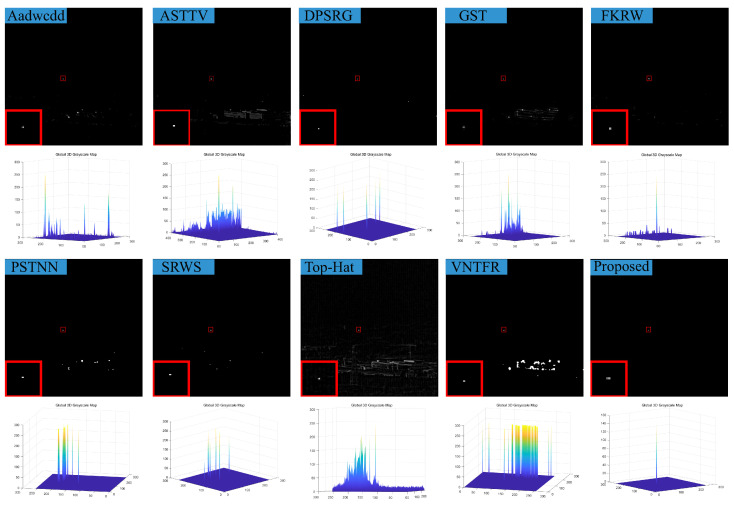
Visual detection effects of compared methods in scene 6.

**Figure 13:**
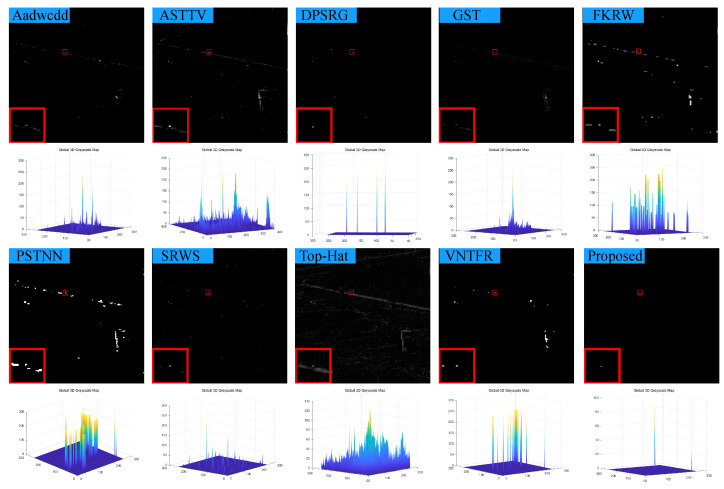
Visual detection effects of compared methods in scene 7.

**Figure 14:**
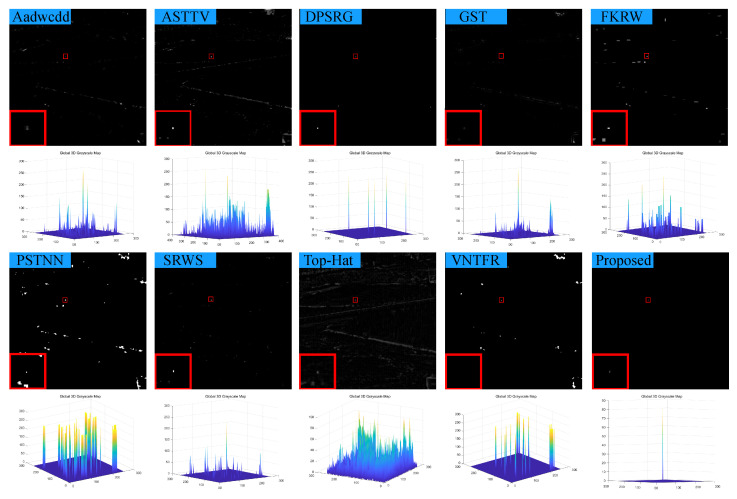
Visual detection effects of compared methods in scene 8.

We compare the visual detection results of the algorithm in this paper with nine algorithms in eight different scenarios and the result shown in Figure 7, Figure 8, Figure 9, Figure 10, Figure 11, Figure 12, Figure 13 and Figure 14. The Aadwcdd [33] algorithm uses the cumulative directional derivative to weight the mean absolute gray difference and eliminate the disadvantage of the decrease in target detection rate when facing strong structured edge backgrounds. However, we can find from Figure 7, Figure 8, Figure 9, Figure 10, Figure 11, Figure 12, Figure 13 and Figure 14 that this algorithm cannot completely suppress the contours and noise in the complex backgrounds, resulting in more background contours and noise in the detection results. The ASTTV [34] algorithm proposes a non-convex approximation tensor rank method that adaptively distributes singular value weights with the help of the Laplace function, and further constrains the background by combining asymmetric spatial–temporal total variation (ASTTV) as a regularization term. However, we can find from Figure 7, Figure 8, Figure 9, Figure 10, Figure 11, Figure 12, Figure 13 and Figure 14 that this algorithm cannot effectively suppress the background when facing strong edge contours and large areas of strong background only by using the global feature information of images. The DPSRG [35] algorithm extracts targets by peak density search and maximum area growth method and can effectively suppress the background. However, we can find from Figure 11 that the DPSRG algorithm cannot separate the target when facing low-energy backgrounds that are submerged in contours, resulting in poorer detection. The GST [36] algorithm models small targets using the second conjugate symmetric derivative of the two-dimensional Gaussian on the directional field. We can find from Figure 9 and Figure 10 that this algorithm cannot detect target points under low-energy target scenes. The FKRW [37] algorithm uses the local contrast descriptor (NLCD) to achieve clutter suppression and target enhancement. We can see from Figure 7, Figure 8, Figure 9, Figure 10, Figure 11, Figure 12, Figure 13 and Figure 14 that NLCD cannot completely inhibit the edge contours under strong edge contours and strong noise conditions. This leads to the appearance of edge residues in the detection results. The PSTNN [19] algorithm and the VNTFR [21] algorithm combine the local prior information and global features of the image to achieve weak target detection. We can find from Figure 8, Figure 9, Figure 10, Figure 11, Figure 12, Figure 13 and Figure 14 that there are a small number of edge contours in the difference map, which is due to the incomplete suppression of the background by the local feature prior information of the PSTNN and VNTFR algorithms, making it impossible to completely constrain the target in the detection model, resulting in a small amount of background residues in the detection results. The SRWS [38] algorithm achieves background suppression by overlapping edge information (OEI). However, we can see from Figure 9 that there are still many edge residues in the detection results under the low-energy target condition. The principle of the top-hat algorithm uses a structural element as a template to perform opening and closing operations on the image to obtain the background model. However, simple opening and closing operations cannot suppress complex backgrounds and noise when facing strong edge contours and noise in complex scenes, leading to more background residues in the detection results. From Figure 7, Figure 8, Figure 9, Figure 10, Figure 11, Figure 12, Figure 13 and Figure 14, we can find that our proposed algorithm has certain advancements compared with the remaining nine algorithms. From the 3D view, we can find that our algorithm completely suppresses the background, eliminates false alarms caused by noise, indicating that our algorithm has excellent noise robustness and background suppression ability.

### 4.4. Quantitative Evaluation

In this section, we quantitatively evaluated our algorithm and the other nine comparative algorithms using SSIM, BSF, SNR, and ROC curves. The results of the calculation of SSIM, BSF, and SNR for our algorithm and nine classic algorithms under six sequential images are shown in Table 1 and Table 2. The bold text in Table 1 and Table 2 represents the maximum value. The parameters used in the experiment were the best values in the corresponding literature. From Table 1 and Table 2, our method can achieve better background estimation compared with other nine advanced algorithms. Moreover, the SNR of our method is the highest in Scene (d), Scene (e), and Scene (f), while in the other scenes, our algorithm can rank in the top three. From Figure 7, Figure 8, Figure 9, Figure 10, Figure 11 and Figure 12, the detection results of SRWS, Aadwcdd, and FKRW have strong target energy, so they have larger SNR values. However, our method is superior to these three algorithms in terms of noise suppression.

**Figure 15:**
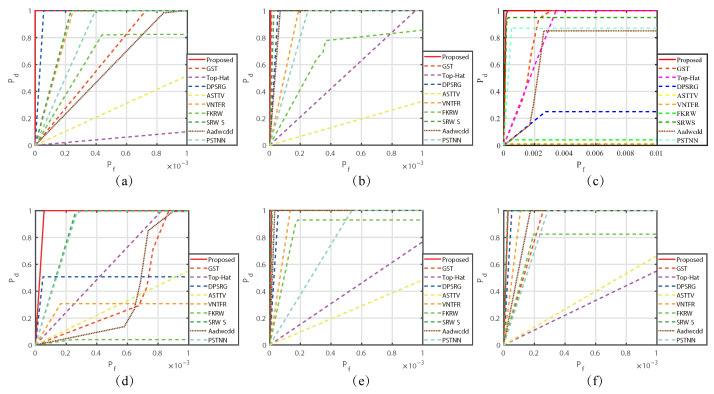
ROC curves of compared methods in six sequences. (**a**–**f**) represent the ROC curves for each of the six sequence scenarios.

We also plotted the ROC curves of each algorithm under the six sequential images, as shown in Figure 15. From Figure 15a–e, we can see that the ROC curve of our algorithm is included in the curves of other algorithms, and the area under the curve is the largest. Therefore, compared with other algorithms, our algorithm has the perfect results.

### 4.5. Algorithm Complexity and Computational Time

Real-time performance and accuracy are two important performance indicators for infrared weak small target detection systems. However, for weak small target detection algorithms, balancing real-time performance and accuracy has become a significant challenge. Traditional spatio-temporal filtering methods have a great advantage in real-time performance due to their simple computation process. However, they often need to improve the accuracy of detecting targets in complex environments with strong edge contours and noise. On the other hand, weak small target detection algorithms based on low-rank and sparse theory have better accuracy in different complex environments. However, due to the use of alternating direction method of multipliers to solve the detection model and complex operations such as SVD decomposition in the solving process, these algorithms often perform poorly in terms of real-time performance. In this paper, a new tensor nuclear norm based on linear transformation is proposed. Through linear transformation in the process of solving this nuclear norm, the original three-dimensional tensor is transformed into a two-dimensional matrix, achieving data dimensionality reduction and effectively reducing the running time of the algorithm. For a sequence tensor image of size $n_{1}\times n_{2}\times n_{3}$, the algorithm proposed in this paper has a complexity of $O\left(n_{1}n_{2}n_{3}^{2}+n_{\left(1\right)}n_{\left(2\right)}^{2}n_{3}\right)$, where $n_{\left(1\right)}=\max\left(n_{1},n_{2}\right)$ and $n_{\left(2\right)}=\min\left(n_{1},n_{2}\right)$. The running time of the proposed algorithm and nine other algorithms on six sequences is shown in Table 3.

## 5. Conclusions

In response to the shortcomings of existing weak target algorithms in an intricate environment, such as low noise robustness, weak background suppression capability, and a high false alarm rate, we propose a low-rank sparse inversion weak target detection method using a joint new paradigm and attention mechanism. In this method, we use an improved tensor nuclear norm to explore the hidden structural information between various modes of the tensor, which can better approximate the tensor rank while reducing time and computational complexity compared to PSTNN, SNN, TNN, etc. We also establish a target sparse constraint algorithm based on self-attention mechanism as a prior information constraint model for target components to achieve better background and target separation and reduce noise and residual background in detection results. Finally, we use optimization algorithms based on ADMM and linear transformation T-SVD to solve the detection model. Based on extensive experimental results in various scenarios, our method demonstrates robustness and outperforms other algorithms in evaluation metrics such as BSF, SSIM, SNR, ROC, etc.

In the sparse target-constrained algorithm based on self-attention mechanism, the image patch size used in this paper is fixed, which has certain limitations when dealing with targets of different sizes. Therefore, if a method can be established to adaptively adjust the image patch size, it will lead to more exciting results. At the same time, we will explore the application of attention mechanism in the time domain and establish a target sparse constraint method based on image temporal–spatial information, which will be integrated into TRPCA model to further improve target detection performance.

## Figures and Tables

**Table 1:** SSIM, BSF, and SNR of nine algorithms under scenarios 1–3.

Method	Scene 1			Scene 2			Scene 3		
	SSIM	BSF	SNR	SSIM	BSF	SNR	SSIM	BSF	SNR
GST	0.9780	48.61	6.09	0.9978	145.02	12.58	0.9818	51.48	−5.09
Top_hat	0.9056	15.55	1.37	0.9568	20.69	8.06	0.9142	11.11	−0.69
DPSR	0.9960	111.31	6.74	0.9984	177.75	7.09	0.9963	115.60	−20.61
ASTTV	0.9945	97.76	3.62	0.9910	77.80	8.63	0.9970	131.25	2.21
VATF	0.9994	296.65	7.72	0.9936	89.58	7.74	0.9941	93.00	−14.30
FKWR	0.9966	122.30	11.56	0.9917	77.92	−0.20	0.9252	25.75	−11.50
SRWS	0.9985	179.71	11.60	0.9993	258.92	12.97	0.9980	156.98	−21.35
Aadcdd	0.9734	44.55	4.56	0.9970	130.68	13.60	0.9818	53.59	−1.70
PSTNN	0.9993	272.29	11.21	0.9996	374.69	12.68	0.9971	132.05	−13.96
Proposed	0.9996	352.68	11.58	0.9998	487.58	13.35	0.9999	750.15	10.10

**Table 2:** SSIM, BSF and SNR of nine algorithms under scenarios 4–6.

Method	Scene 4			Scene 5			Scene 6		
	SSIM	BSF	SNR	SSIM	BSF	SNR	SSIM	BSF	SNR
GST	0.9939	87.87	−0.03	0.9989	214.07	11.52	0.9930	85.34	5.86
Top_hat	0.9084	0.91	2.45	0.9554	22.75	5.94	0.9687	30.43	2.18
DPSR	0.9935	87.83	5.84	0.9986	188.34	7.01	0.9979	153.16	6.84
ASTTV	0.9970	130.10	3.17	0.9975	143.75	7.06	0.9964	120.51	5.37
VATF	0.9990	228.44	−18.20	0.9987	192.82	11.40	0.9915	77.26	1.67
FKWR	0.9523	32.39	3.60	0.9985	183.79	13.02	0.9984	177.42	12.01
SRWS	0.9978	151.57	2.23	0.9993	270.59	11.99	0.9994	297.31	11.23
Aadcdd	0.9853	58.84	−2.33	0.9983	173.45	9.52	0.9946	96.82	6.74
PSTNN	0.9980	160.27	4.15	0.9993	264.60	11.64	0.9983	171.51	7.97
Proposed	0.9997	405.94	10.91	0.9997	443.82	13.57	0.9996	363.23	10.82

**Table 3:** Comparison of the running time (in seconds) of the ten methods.

Methods	Sequence 1	Sequence 2	Sequence 3	Sequence 4	Sequence 5	Sequence 6
Aadcdd	0.031775	0.025940	0.025343	0.026590	0.025896	0.025677
ASTTV	2.025396667	1.81317567	1.80657	1.730859	1.74083933	1.752016
DPSR	0.360631	0.266406	0.259233	0.256414	0.268786	0.260065
GST	0.034531	0.021335	0.021900	0.021537	0.023158	0.022639
FKWR	0.122541	0.108435	0.094564	0.081714	0.068038	0.113238
PSTNN	0.406764	0.227669	0.229637	0.209995	0.228107	0.209519
SRWS	1.926410	1.956192	1.777600	1.659314	1.859583	1.714700
Top_hat	0.248881	0.155434	0.289018	0.282346	0.282783	0.289122
VATF	0.912477	0.926117	0.912015	0.909593	0.918572	0.882623
Proposed	0.009312727	0.009789	0.01751591	0.02709809	0.026807	0.019232818

## Abbreviations

The abbreviations are used in this manuscript as following:

Aadwcdd	Absolute average difference weighted by cumulative directional derivatives
ASTTV	Asymmetric Spatial–Temporal Total Variation
DPSRG	Density Peaks Searching and Maximum-Gray Region Growing
GST	Generalized Structure Tensor
FKRW	Facet Kernel and Random Walker
PSTNN	Partial sum of tensor nuclear norm
VNTFR	via Nonconvex Tensor Fibered Rank
SRWS	Self-Regularized Weighted Sparse
SSIM	Structural Similarity Image
SNR	Signal-to-Noise Ratio
BSF	Background Suppression Factor
